# Ectoparasites and Endoparasites of the Clark’s Lizard, *Darevskia clarkorum* (Squamata, Lacertidae), Collected from Different Provinces of the Black Sea Region of Türkiye

**DOI:** 10.2478/helm-2026-0001

**Published:** 2026-04-27

**Authors:** Y. CIVIL, H. S. YILDIRIMHAN, A. M. REDHWAN, U. BÜLBÜL, S. SARIKURT, A. A. HASSAN

**Affiliations:** Department of Biology, Faculty of Science and Literature, Bursa Uludag University, 16059, Nilufer, Bursa, Türkiye fiille240@gmail.com; Department of Biology, Faculty of Science, Karadeniz Technical University, 61080, Trabzon, Türkiye

**Keywords:** Clark’s Lizard, Cestoda, Nematoda, Acanthocephala, Arthropoda, Türkiye

## Abstract

A total of 61 specimens (26♀♀, 31 ♂♂, and 4 juveniles) of *Darevskia clarkorum* (Darevsky & Vedmederja, 1977), collected between 2000 and 2005 from five provinces of Türkiye (Ordu, Giresun, Trabzon, Rize, and Artvin) and archived as museum material, were subjected to helminthological examination. As a result of the examinations conducted, 2 Cestoda (*Nematotaenia tarentolae, Oochoristica tuberculata*), 4 Nematoda (*Strongyloides darevskyi, Oswaldocruzia filiformis, Skrjabinodon medinae, Agamospirura* sp.), and 1 Acanthocephala (*Centrorhynchus* sp. larvae) species were identified in the host. In addition, an ectoparasite species in the arthropod belonging to the order Ixodida (*Ixodes ricinus*) was encountered. Except for *Ixodes ricinus*, all parasite species have been recorded for the first time in *D. clarkorum* in the literature. In this respect, the study makes an important contribution to both the species’ parasite fauna and the herpetofauna of Türkiye.

## Introduction

Türkiye has a rich reptile fauna with 141 species. Lizards (Lacertilia), which constitute a significant portion of these species, are represented by 70 species across 9 families in the country and account for approximately 49.6 % of the total reptile fauna. (Yaşar *et al*., 2021). Various studies have been conducted in Türkiye to determine the parasite fauna on 38 lizard species to date (Schad *et al*., 1960; Tınar, 1982; Tınar, 1983; Saygı & Olgun, 1993; Yıldırımhan *et al*., 2006; Gürelli *et al*., 2007; Yıldırımhan *et al*., 2008; Düşen *et al*., 2010; Yıldırımhan *et al*., 2011; Düşen *et al*., 2013; İncedoğan *et al*., 2014; Birlik *et al*., 2015; Roca *et al*., 2015a; Roca *et al*., 2015b; Roca *et al*., 2016; Düşen *et al*., 2016; Birlik *et al*., 2016; Düşen *et al*., 2017; Birlik *et al*., 2017a; Birlik *et al*., 2017b; Birlik *et al*., 2018a; Birlik *et al*., 2018b; Yıldırımhan *et al*., 2018; Yıldırımhan & Sümer., 2019; Yıldırımhan *et al*., 2020a; Yıldırımhan *et al*., 2020b; Birlik *et al*., 2020; Yıldırımhan *et al*., 2021; Yıldırımhan *et al*., 2022; Birlik, 2022; Sümer *et al*., 2023; Yıldırımhan *et al*., 2023; Birlik *et al*., 2024; Hastürk *et al*., 2025a; Hastürk *et al*., 2025b; Yıldırımhan *et al*., 2025).

The species we will examine in this study, *Darevskia clarkorum* (Darevsky & Vedmederja, 1977), is a medium-sized lizard with a brown back in males and a green back in females. This species is distributed in the Middle East and Western Asia. It is found in our neighboring region of Georgia (Adzharia region) and in Türkiye along the Black Sea coast (from Artvin to Hopa and Giresun). Previous research has demonstrated that this species occurs not only in coastal areas of the region but also in inland localities such as Gümüşhane (Akocak) (Kurnaz & Kutrup, 2018). The Clark’s lizard, whose numbers are steadily declining, is classified as endangered (EN) according to the IUCN Red List (Ilgaz, 2007).

**Fig. 1.**
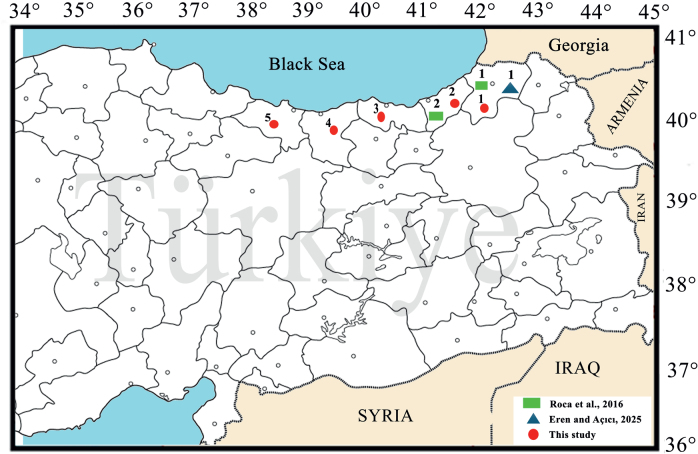
Localities where *Darevskia clarkorum* was collected.

Parasitological data on *D. clarkorum* are quite limited in the literature. In previous studies of this species, only one Nematoda species has been reported among endoparasites (Roca *et al*., 2016), whereas seven nematode species have been reported among ectoparasites (Jabbarpour, 2016; Eren & Açıcı, 2025) (Fig. 1). This study constitutes the first comprehensive assessment of both endoparasites and ectoparasites in *D. clarkorum* and aims to enhance current knowledge of the parasite diversity of Türkiye.

## Material and Methods

A total of 61 specimens (26 ♀♀, 31 ♂♂, and 4 juveniles) of *Darevskia clarkorum*, collected between 2000 and 2005 from the provinces of Ordu (3♀♀, 5♂♂, 1J), Giresun (3♀♀, 2♂♂,1J), Trabzon (5♀♀, 6♂♂), Artvin (6♀♀, 6♂♂), and Rize (9♀♀, 12♂♂, 2J)in the Black Sea Region of Türkiye (Fig. 1, Table 2), have been stored as museum material under appropriate conditions (70 % alcohol) at the Zoology Laboratory of Karadeniz Technical University. The specimens were subsequently transferred to the Zoology Laboratory of the Department of Biology at Bursa Uludağ University for parasitological examination. All information about the specimens (collection site, sex, etc.) was recorded before dissection. Before dissection, the body’s external surface was examined under a stereomicroscope to detect the presence of ectoparasites and any parasites found were collected in bottles filled with 70 % alcohol for species identification. Subsequently, dissection of the lizard specimens was initiated by making a longitudinal incision along the ventral part of the body to expose the internal organs. Under a stereo microscope, the animals’ body cavities were first examined, and then all internal organs were removed and placed in separate petri dishes. The parasites found by carefully examining the animals’ internal surfaces, followed by their hearts, livers, esophagus, stomachs and intestines, were stored in bottles containing 70 % alcohol until their species could be identified. During preparation, Cestoda specimens were stained with Ferrous Aceto-Carmine (Georgiev *et al*., 1986). They were sealed with Entellan®. Samples of Nematoda were prepared as permanent mounts by covering them with lactophenol without any staining. The parasite species were identified using sources by Yamaguti (1961), Sharpilo (1976), Petter and Quentin (1976), Özkan (1978), and Schmidt (1986). Prevalence, average density, and abundance values were calculated according to Bush *et al*. (1997). The identified parasite species are stored in the Parasitology Collection of the Zoology Museum at Bursa Uludağ University.

### Ethical Approval and/or Informed Consent

Specimens of *Darevskia clarkorum* used in this study were collected between 2000 and 2005, a period when institutional or national animal ethics committees had not yet been established in Türkiye. Therefore, no ethical approval was required at the time of collection. All specimens have since been preserved as museum material at the Zoology Laboratory of Karadeniz Technical University under standard conditions.

### Results

Of the 61 specimens of *D. clarkorum*, 34 (55.7 %) were infected with a parasite species. No parasites were found in 26 (44.3 %) specimens. A total of 108 individuals from 8 parasite species were found in the host species. In infected lizards, 2 individuals of *Oochoristica tuberculata* (Rudolphi, 1819) were found in two hosts, 1 individual of *Nematotaenia tarentolae* Lopez-Neyra, 1944 in one host, 25 individuals of *Strongyloides darevskyi* Sharpilo, 1976 in eight hosts, 1 individual of *Oswaldocruzia filiformis* (Goeze, 1782) Skrjabin & Schultz, 1952 in one host, 65 individuals of *Skrjabinodon medinae* (García-Calvente, 1948) in twenty-seven hosts, 2 individuals of *Agamospirura* sp. Henry & Sisoff, 1913 in one host, 9 larvae of *Centrorhynchus* sp. Lühe, 1911, in four hosts, and 3 individuals of *Ixodes ricinus* (Linnaeus, 1758) (larval and nymphal stages) in two hosts.

The parasite species *N. tarentolae, O. tuberculata, S. darevskyi, O. filiformis, S. medinae, Agamospirura* sp., and *Centrorhynchus* sp. (larval stage) were recorded for the first time from this host species. In addition, the provinces of Ordu and Giresun were examined parasitologically for the first time with respect to this host. The infection values of the parasite species found are given in Table 1. The studies revealed that the highest prevalence and abundance were observed in *S. medinae*, while the highest average density was observed in *S. darevskyi*.

**Table 1.** Prevalence, mean intensity, mean abundance, localization, and intensity range (min–max) of helminth species recorded from *Darevskia clarkorum*.

Parasite species	Site of infection	Prevalence (%)	Mean intensity	Mean abundance	Min-Max
*Nematotaenia tarentolae*	Intestine	1.6	1	0.01	1
*Oochoristica tuberculata*	Intestine	3.2	1	0.03	1
*Strongyloides darevskyi*	Intestine	13.1	3.1	0.4	1 – 11
*Oswaldocruzia filiformis*	Intestine	1.6	1	0.01	1
*Skrjabinodon medinae*	Intestine	44.2	2.4	1.06	1 – 7
*Agamospirura* sp.	Intestine	1.6	2	0.03	2
*Centrorhynchus* sp. larvae	Body cavity	6.5	2.2	0.1	1 – 4
*Ixodes ricinus*	Skin (external surface)	3.2	1.5	0.05	1

### Discussion

To our knowledge, three parasitological studies have been conducted worldwide on *D. clarkorum*, and the present study represents the fourth investigation of this species. The first study was conducted by Roca *et al*. (2016) on 29 individuals (13♂, 16♀) collected from the provinces of Rize and Artvin, and a single endoparasite species, *Skrjabinodon saxicolae* Sharpilo, 1961 (13.8 %), was detected in infected individuals. In the current study, unlike the previous study, three new provinces (Ordu, Giresun, and Trabzon) were added to the sampling locations, and a total of seven new endoparasite species were added to the literature.

Data on the ectoparasite fauna of *D. clarkorum* are restricted to two studies in the literature. Jabbarpour (2016) reported the presence of *Lacertacarus similis* Schluger and Vasilieva 1977 and *Ophionyssus saurarum* (Oudemans, 1901) in two of nine *D. clarkorum* specimens collected from the provinces of Artvin, Rize, and Trabzon. In a subsequent study, Eren and Açıcı (2025) identified six ectoparasite species (*Ixodes ricinus* (Linnaeus, 1758), *Lacertacarus callosus* (Schluger 1966), *L. similis, Odontacarus hushchai* Kudryashova, 1994, *Odontacarus naumovi* Kudryashova & Rybin, 1974 and *Odontacarus saxicolis* Schluger, Hushcha & Kudryashova, 1965) in 28 specimens obtained from Artvin. The detection of *I. ricinus* in the present study is consistent with previous findings and suggests continuity in the species’ ectoparasite fauna, particularly among tick taxa. These findings indicate that *D. clarkorum* may serve as a suitable host for certain ectoparasite species.

In the helminths identified in this study, *N. tarentolae* lacks distinct external segmentation; only the acraspedote segment boundaries at the posterior end of mature individuals are discernible. The skolex shows a slight constriction behind the suckers. The fact that each paraterine organ contains only one egg is considered a characteristic feature of the species (Sharpilo, 1976). This parasite is commonly reported in various lizard species (Roca *et al*., 1985). It exhibits an indirect (dixenous) life cycle, in which lizards act as definitive hosts, while insects belonging to the order Coleoptera are considered probable intermediate hosts (Carretero *et al*., 2006). The first record in Türkiye was reported by Roca *et al*. (2015b) in *D. rudis* (Bedriaga, 1886). It has also been reported in *D. parvula* (Lantz & Cyrén, 1913) and *D. valentini* (Boettger, 1892) (Roca *et al*., 2016). This study represents the fourth record in Türkiye.

*Oochoristica tuberculata* is characterized by a hookless scolex with four suckers, a short neck, and acraspedote segments. Mature segments contain more than 30 (30 – 40) testes. This species is widely distributed across Europe, North Africa, and Central Asia. It is widely distributed among reptiles and is known to be an obligate parasite of lizards. The life cycle of this genus typically involves intermediate hosts such as insects and spiders (Dollfus, 1954; Della Santa, 1956). The first record in Türkiye was made on *Laudakia caucasia* (Eichwald, 1831) (Yıldırımhan *et al*., 2006). Subsequent studies have also reported species belonging to the genus *Darevskia*: *D. valentini* (Birlik *et al*., 2018b); *D. derjugini* (Nikolsky, 1898); *D. parvula, D. raddei* (Boettger, 1892); and *D. rudis* and *D. unisexualis* (Darevsky, 1966) (Birlik, 2022). This study represents the seventh record of *Darevskia* species in Türkiye.

*Strongyloides darevskyi* bears transverse lines on the cuticle that are almost imperceptible. The mouth opening is round, and the lips are indistinct; there are four small submedial protrusions at the anterior end. In the lower third of the body, there is a vulva with slightly protruding lips. Among the *Strongyloides* genus of nematodes recorded among reptiles, this species has been identified as the smallest species (Sharpilo, 1976). *S. darevskyi* is, in fact, a true *Darevskia* specialist since it has been recorded only from species of this genus (Roca *et al*., 2016). The first record in Türkiye was made by Roca *et al*. (2015b) on *D. rudis*. It has also been reported on *D. armeniaca* (Méhely, 1909) (Roca *et al*., 2016), *D. parvula, D. raddei, D. rudis* and *D. valentini* (Birlik, 2022). This study represents the seventh record of *Darevskia* species in Türkiye.

*Oswaldocruzia filiformis* is a small and slender nematode with a wide global distribution. Male individuals are characterized by spines that are divided into three parts at the distal end and lack extra branching in the proximal region (Birlik, 2022). The spicule structures are elongated, each nearly equal in length and surrounded by a thin membrane. Additionally, the caudal bursa possesses distinctive morphological structures such as variable dorsal rays. Females have significantly longer bodies than males. Their eggs have an oval shape, and the posterior end of the body ends in a pointed manner (Kirillova *et al*., 2020). The life cycle of *O. filiformis* is direct. The principal hosts, including amphibians and lizards, become infected by ingesting the infective larvae with their food (Svitin *et al*., 2016). The first record of the *O. filiformis* species in Türkiye was made by Schad *et al*. (1960) on *Bufo regularis* Reuss, 1833 and *Rana macrocnemis* Boulenger, 1885. In subsequent years, this species has also been reported in some species within the genus *Darevskia*. These include *D. rudis* (Roca *et al*., 2015b; Birlik *et al*., 2018a), *D. derjugini* and *D. parvula* (Birlik, 2022). This study represents the fourth record of the *Darevskia* species in Türkiye.

*Skrjabinodon medinae* was first described by Garcia Calvente (1948) as *Pharyngodon medinae*. Subsequently, Specian and Ubelaker (1974) assigned *P medinae* to *Skrjabinodon* because it lacked caudal alae and possessed a single pair of sessile pre-cloacal papillae. (Hornero & Roca, 1992). The life cycle of *S. medinae* has not been investigated; however, members of the family Pharyngodonidae are known to have a strictly monoxenous life cycle (Anderson, 2000). It was first recorded in Türkiye in 2011 by Yıldırımhan *et al*. (2011) in *Lacerta trilineata* Bedriaga, 1886. In our country, the following species of lizards in the genus *Darevskia* have been recorded: *D. rudis* (Birlik *et al*., 2018a), *D. valentini* (Birlik *et al*., 2018b), *D. parvula*, and *D. unisexualis* (Birlik, 2022). This study represents the fifth record of *Darevskia* species in Türkiye.

*Agamospirura* sp. are whitish nematodes with an enticulate structure and indistinct transverse stripes on their surface. Two well-developed lateral cuticular aleas extend along the body. Four large cephalic papillae are located in the head region (Moravec *et al*., 1987). Although the complete life cycle of *Agamospirura* species has not been fully elucidated, available evidence suggests that they follow the typical spirurid developmental pattern. Spirurid nematodes are characterized by an indirect (heteroxenous) life cycle involving arthropod intermediate hosts, most commonly insects. Reptiles, including lizards and snakes, may serve as paratenic hosts, in which larval stages can persist without further development. (Anderson, 2000; Dollfus, 1954). Although there are few records worldwide, it has been detected in different reptile species: *Lacerta agilis* Linnaeus, 1758, *Natrix natrix* (Linnaeus, 1758), *Coronella austriaca* Laurenti, 1768, and *Vipera berus* (Shevchenko, 1963). There are no records of this species in Türkiye’s fauna. Therefore, this is a new locality record for *Agamospirura* sp. in Türkiye. *Centrorhynchus* (Polymorphida: Centrorhynchidae) is characterized by a generally cylindrical proboscis with a slightly swollen neck region armed with inwardly curved hooks bearing 28 – 30 longitudinal rows, each forming 20 – 23 hooks (Sharpilo, 1976; Komorová *et al*., 2015). It is a genus of acanthocephalans primarily found in predatory birds belonging to the orders Falconiformes and Strigiformes. However, a few species have also been reported from mammals and reptiles. *Centrorhynchus*, which comprises approximately 90 species, is one of the most common and species-rich genera of acanthocephalans parasitizing birds of prey (Choi *et al*., 2010; Golvan, 1994; Richardson & Nickol, 1995). Intermediate hosts include terrestrial isopods and various insect species. Additionally, amphibians, reptiles, and mammals are among the paratenic hosts that contribute to the parasite’s transmission to birds (Buron & Golvan, 1986). In the study, *Centrorhynchus* sp. was found in the larval stage and attached to the outer surface of the intestine. The species-level identification of acanthocephalans belonging to the genus *Centrorhynchus* is only possible based on adult forms. *Centrorhynchus* larvae have previously been recorded in lizards from Türkiye, specifically in *Apathya cappadocica* (Werner, 1902) (Birlik *et al*., 2015), *Acanthodactylus harranensis* Baran, Kumlutas, Lanza, Sindaco, Avci & Crucitti, 2005 (Düşen *et al*., 2016), and *Anatololacerta anatolica* (Werner, 1900) (Hastürk *et al*., 2025a). Additionally, this genus has been identified at the species level (*Centrorhynchus aluconis* (Müller, 1780)) in *Asaccus elisae* (Werner, 1895) (Yıldırımhan *et al*., 2018). *Centrorhynchus* sp. represents the first record for this host species in Türkiye, having been detected on *D. clarkorum*.

*Ixodes ricinus* is distributed across the Western Palearctic, particularly in moist habitats with deciduous, coniferous, or mixed forests (Estrada-Peña *et al*., 2018). Larvae and nymphs typically feed on small mammals, birds and reptiles, while adults are more parasitic on large mammals (Nicholson *et al*., 2009; Medlock *et al*., 2013). In this study, individuals at the larval and nymphal stages were detected on the host species’ skin surface. In Türkiye, the species *I. ricinus* has previously been found in various reptile species, particularly turtles (Bakirci, 2016; Eren *et al*., 2023; Jabbarpour, 2016; Keskin *et al*., 2012; Keskin *et al*., 2013; Kirecci *et al*., 2013; Yaman & Zerek, 2016; Yilmaz *et al*., 2013). In addition, records of this tick are also present in some species belonging to *Darevskia*. The presence of *I. ricinus* has previously been reported in Türkiye on *D. adjarica* (Darevsky & Eiselt, 1980), *D. clarkorum, D. derjugini, D. obscura* (Lantz & Cyrén, 1936) (Eren & Açıcı, 2025) and *D. rudis* (Keskin *et al*., 2012). This study presents the second record of *I. ricinus* on *D. clarkorum*.

In the present study, parasites were detected in 17 of 26 female *D. clarkorum* (65 %), 27 of 31 males (87 %), and 2 of 4 juvenile individuals (50 %) (Table 2). Overall, parasite prevalence was higher in males than in females and juveniles.

**Table 2.** Distribution of parasite species among lizards collected from different localities in the Eastern Black Sea Region, categorized by host sex.

Parasite species	Number of infected lizard ♂, ♀ and J															Total Number of infected		
	Ordu (n=9)			Giresun (n=6)			Trabzon (n= 11)			Rize (n=23)			Artvin (n=12)			lizard (n=61)		
	♀3	♂5	J1	♀3	♂2	J1	♀5	♂6	J0	♀9	♂12	J2	♀6	♂6	J0	♀26	♂31	J4
*Nematotaenia tarentolae*	-	-	-	-	-	-	-	1	-	-	-	-	-	-	-	-	1	-
*Oochoristica tuberculata*	-	-	-	-	-	-	1	-	-	-	-	-	-	1	-	1	1	-
*Strongyloides darevskyi*	-	3	-	-	-	-	-	-	-	1	4	-	-	-	-	1	7	-
*Oswaldocruzia filiformis*	-	-	-	-	-	-	-	-	-	1	-	-	-	-	-	1	-	-
*Skrjabinodon medinae*	-	1	-	1	2	1	4	3	-	3	7	1	3	1	-	11	14	2
*Agamospirura* sp.	-	-	-	-	-	-	-	-	-	1	-	-	-	-	-	1	-	-
*Centrorhynchus* sp. larvae	-	-	-	-	-	-	-	-	-	2	2	-	-	-	-	2	2	-
*Ixodes ricinus*	-	-	-	-	-	-	-	-	-	-	1	-	-	1	-	-	2	-

* ♂Male, ♀Female and J juvenile

In the literature, the observation that parasite burden is higher in male lizards in some cases can be explained by two main ecological and physiological factors. First, males tend to have larger home ranges and higher mobility, particularly during the breeding season (McCurdy *et al*., 1998). This increased movement may increase the likelihood of contact with environmentally infective parasite stages, thereby increasing the risk of parasitism in males. Second, the immunocompetence–handicap hypothesis suggests that elevated testosterone levels may suppress immune function, rendering male individuals more susceptible to parasitic infections (Folstad & Karter, 1992). Experimental studies supporting this hypothesis have shown that testosterone administration to male *L. agilis* increases the tick burden (Olsson *et al*., 2000).

Our study revises the parasites of *D. clarkorum*, increasing the number of endo and ectoparasite species from eight to fifteen. Compared with previous studies, it is noticeable that increasing the number of localities and samples collected also increases parasite diversity. Therefore, it is expected that studies that increase the number of samples collected from various localities in the coming years will also identify more helminth species. Furthermore, given that *D. clarkorum* is an endangered species and that future studies may be limited by insufficient specimen availability, this study provides highly valuable data.
